# Segmental Bronchial Allergen Challenge Elicits Distinct Metabolic Phenotypes in Allergic Asthma

**DOI:** 10.3390/metabo12050381

**Published:** 2022-04-22

**Authors:** Yanlong Zhu, Stephane Esnault, Ying Ge, Nizar N. Jarjour, Allan R. Brasier

**Affiliations:** 1 Department of Cell and Regenerative Biology, School of Medicine and Public Health (SMPH), University of Wisconsin-Madison, Madison, WI 53705, USA; yzhu353@wisc.edu (Y.Z.); ying.ge@wisc.edu (Y.G.); 2 Human Proteomics Program, School of Medicine and Public Health (SMPH), University of Wisconsin-Madison, Madison, WI 53705, USA; 3 Division of Allergy, Pulmonary and Critical Care Medicine, Department of Medicine, School of Medicine and Public Health (SMPH), University of Wisconsin-Madison, Madison, WI 53705, USA; sesnault@medicine.wisc.edu; 4 Institute for Clinical and Translational Research (ICTR), University of Wisconsin-Madison, Madison, WI 53705, USA

**Keywords:** allergic asthma, saturated fatty acid synthesis, segmental bronchial antigen provocation, metabolomics, phenotype

## Abstract

Asthma is a complex syndrome associated with episodic decompensations provoked by aeroallergen exposures. The underlying pathophysiological states driving exacerbations are latent in the resting state and do not adequately inform biomarker-driven therapy. A better understanding of the pathophysiological pathways driving allergic exacerbations is needed. We hypothesized that disease-associated pathways could be identified in humans by unbiased metabolomics of bronchoalveolar fluid (BALF) during the peak inflammatory response provoked by a bronchial allergen challenge. We analyzed BALF metabolites in samples from 12 volunteers who underwent segmental bronchial antigen provocation (SBP-Ag). Metabolites were quantified using liquid chromatography-tandem mass spectrometry (LC–MS/MS) followed by pathway analysis and correlation with airway inflammation. SBP-Ag induced statistically significant changes in 549 features that mapped to 72 uniquely identified metabolites. From these features, two distinct inducible metabolic phenotypes were identified by the principal component analysis, partitioning around medoids (PAM) and k-means clustering. Ten index metabolites were identified that informed the presence of asthma-relevant pathways, including unsaturated fatty acid production/metabolism, mitochondrial beta oxidation of unsaturated fatty acid, and bile acid metabolism. Pathways were validated using proteomics in eosinophils. A segmental bronchial allergen challenge induces distinct metabolic responses in humans, providing insight into pathogenic and protective endotypes in allergic asthma.

## 1. Introduction

Allergic asthma is a heterogeneous disease that arises from complex epigenetic-environmental interactions. Multiple pathophysiological pathways interact to produce a syndrome characterized by episodic airway obstruction, nonspecific bronchial hyper-responsiveness, and inflammation [1]. In patients with severe or difficult to treat asthma, acute decompensations are responsible for decreased quality of life [2], unscheduled healthcare delivery increasing costs [3], and may accelerate structural remodeling [3,4,5], impacting long-term reduction in lung function [4]. In patients with allergic asthma, episodes of decompensation can be provoked by exposure to aeroallergens. 

The airway mucosa in patients with allergic asthma is enriched in transitioned epithelial cells, IgE-bearing mast cells, basophils, eosinophils, and Th2 lymphocytes [1,5]. In this context, luminal aeroallergens directly activate innate responses in epithelium [6] as well as cross-link IgE that triggers degranulation and release of proinflammatory mediators, such as histamine, tryptase, leukotrienes, lipid mediators, and Th2 cytokines [7]. These mediators act rapidly to cause smooth muscle contraction, increase vascular permeability, and mucus secretion. In over half of individuals, the immediate allergic reaction is followed by a more sustained inflammation, the late-phase response [8], characterized by recruitment of effector Th2 lymphocytes and CD45 expressing leukocytes, with bronchoconstriction, resulting in a reversible fall in FEV_1_ and clinical decompensation [9]. At the individual level, the age of onset, the multiple different types of exacerbators, variable role of innate inflammation [10], and distinct roles of Th17/Th2 T lymphocyte populations suggest that distinct pathophysiological processes contribute to the syndrome [11]. 

Regarding allergic asthma, researchers have focused on identifying disease-relevant biomarkers in moderate and severe asthma, including in the severe asthma research program [12,13] and the Unbiased Biomarkers for the Prediction of Respiratory Disease Outcome (U-BIOPRED) project [14]. These studies have converged on several main asthma phenotypes that have distinct clinical features distinguished by differences in onset and types of inflammation (eosinophilic vs. non) [11]. However, these phenotypes contain multiple subgroups and overlap substantially [11,15], indicating that more advances in endotype identification will be impactful for informing precision medicine in asthma. 

One such approach involves unbiased analyses of allergic asthmatics in the peak of the inflammatory response to provide information on the cellular processes controlling clinical exacerbations. To this end, we have employed segmental bronchoprovocation with an antigen (SBP-Ag) that has led to the understanding of secreted protein factors promoting the inflammatory response [16,17]. Knowing that activation produces profound metabolic compensation in epithelial cells [18], lymphocytes [19], and eosinophils [20], we sought to apply data-dependent metabolomics profiling to controlled allergen exposure leading to peak inflammatory response. We present unbiased identification of two distinct metabolic profiles in a group of allergic asthmatics that were otherwise indistinguishable under basal conditions. A total of 72 index metabolites were analyzed for pathway enrichment, where upregulation of saturated fatty acid synthesis and mitochondrial beta oxidation of saturated fatty acids were observed. Fatty acid biosynthetic pathways were validated by proteomics in eosinophils. We propose that an analysis of inducible phenotypes may provide novel insight into latent asthma endotypes. 

## 2. Results

### 2.1. Demographics

**Figure 1:**
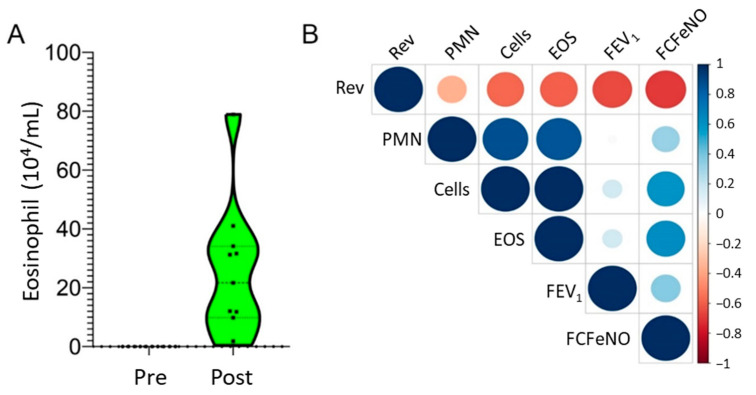
Phenotypic analysis of allergic asthma. (**A**) BALF eosinophil counts before and after SBP-Ag. Data are presented as a violin plot to demonstrate response variability. (**B**) Correlation between clinical demographic variables. Correlogram of pairwise comparison for study group demographics (Table 1). Note that change in FeNO is inversely correlated to albuterol reversibility and eosinophil (EOS) numbers are correlated with total cells and neutrophils (PMNs). Abbreviations: FCFeNO, fold change of FeNO; Rev, albuterol reversibility.

Volunteers in this study averaged 26 ± 5 years of age and resting predicted FEV_1_ 95 ± 16% (Table 1). After the allergen challenge, BAL eosinophil counts rose to 23 ± 21.6 × 10^4^ from 0.01 ± 0.01 × 10^4^ cells/mL, constituting 17.7 to 80.6% of total BAL cells (Figure 1A). There was marked heterogeneity in the eosinophil count across patients, despite receiving a standard allergenic dose by PC_20_. To further understand the relationships of the clinical phenotypic data, Pearson correlations were performed of physiological measurements and measures of cellular inflammation in the BALF and plotted as a correlogram (Figure 1B). Consistent with previous knowledge, the correlogram indicates a strong positive relationship between total cellularity, eosinophils, and polymorphonuclear leukocytes (PMNs, neutrophils). By contrast, a strong inverse relationship of FEV_1_ with albuterol reversibility and a fold change in FeNO (FCFeNO) was observed, whereas FCFeNO was positively correlated with total cells and eosinophil numbers (Figure 1B). 

### 2.2. Differentially Regulated Metabolites

Metabolites in cell-free BALF from pre- and post-SBP-Ag were processed and identified by LC–MS/MS. The complete mass list is shown in Supplemental Table S1. Significantly changed analytes were identified by pairwise statistical analysis of microarray (SAM). This pairwise analysis maximized the sample power of the pairwise experimental design and reduced impact of individual variability [21]. Moreover, SAM accommodated the nonparametric distribution of metabolite expression characteristic of these pathways. A total of 549 metabolites whose abundances were significantly changed were identified by their wide deviations of the expected vs. observed abundances using a high stringency cut-off of Δ = 1.0 (dashed line in Supplemental Figure S1). Of these, SBP-Ag increased the abundance of 428 metabolic features and reduced the expression of 121 metabolic features (Supplemental Table S2). From these statistically significant metabolic features, 72 metabolites were uniquely identified (Supplemental Table S3).

### 2.3. Global Metabolomics Identify Distinct Metabolite Responses

**Figure 2:**
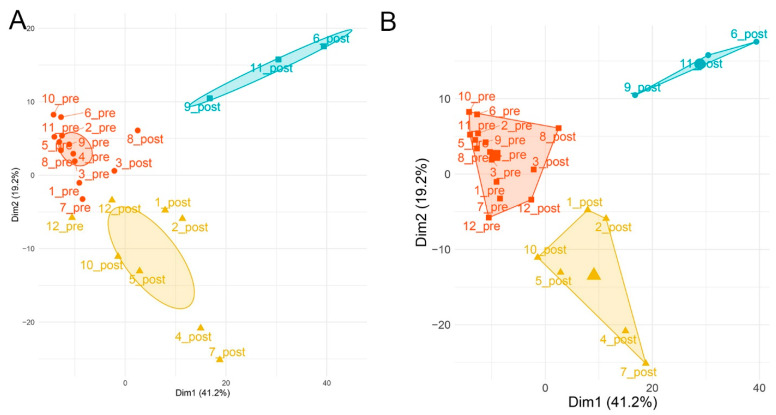
Global expression analysis. (**A**) Partition around medoids (PAM). The abundance of 549 significant metabolites were subjected to PAM. Subjects before challenge were grouped into one cluster (orange). After challenge, patients were grouped into two distinct clusters (clusters indicated by blue- and yellow-colored ellipses). (**B**) K-means clustering. K-means clustering of significant metabolites. The mean of each centroid is represented by the large-filled symbol. Note that SBP-Ag produces similar divergence of subjects into two classes with subject no. 12 being indeterminate.

We next explored the expression patterns of 549 metabolites whose abundance was statistically different between pre- and post-SBP-Ag. Using the principal components analysis (PCA), we observed that the metabolites placed the volunteers into two distinct groupings after SBP-Ag (Supplemental Figure S2). To confirm this surprising finding, we analyzed the results using partitioning around medoids (PAM), a well-established partitioning method that is robust to outlier influence and leads to better interpretation, as the cluster centers are data points (aka “medoids”) and additional cluster members are assigned by the nearest method estimation [22,23]. From this analysis, a similar partitioning was identified in the post-SBP-Ag samples, patients 9, 11, and 6 all representing post-challenge samples were grouped together, and patients 1, 2, 4, 5, 7, and 10, grouped together, also representing post-allergen challenge samples, were separated by the second dimension (Figure 2A). To confirm, K-means clustering was applied using the optimal clustering size by the elbow method (minimum of three clusters Supplemental Figure S3). A similar grouping was obtained, where the pre-SBP-Ag samples where closely related, but the post-SBP-Ag samples were separated by the second dimension and found in two separate groups (Figure 2B). Collectively, the data from the PCA, PAM, and k-means clustering led us to conclude that the metabolomics patterns produced at least two distinct subject groupings. 

**Figure 3:**
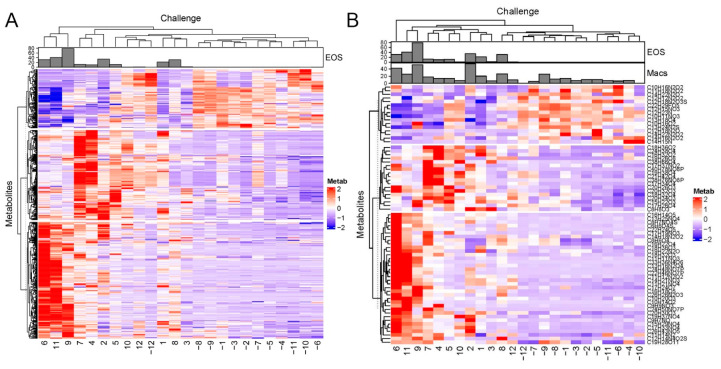
Hierarchical clustering. (**A**) Hierarchical cluster for 549 significant metabolites. Metabolite abundance was expressed by feature intensity and normalized by the z-score. Metabolites are rows, patients are columns. Patient IDs indicated by “-” are baseline (pre) challenge. Each patient is annotated by the number of eosinophils in the BALF sample (number × 10^4^)/mL shown in the bar graph at top. Note that three major clusters of patients (columns) were separated by pre- vs. post-treatment. (**B**) Hierarchical cluster with 72 identified metabolites. At the top is the annotation of eosinophil numbers (EOS) and macrophage numbers (Macs) in each sample. Note that, (1) the dendrograms are very similar; (2) volunteers subjected to SBP-Ag are clearly separated from the pre-challenge (columns); and (3) two distinct groupings of post-SBP-Ag are produced. Chemical formulas are shown on the right.

To further explore the relationships of metabolite profiles to one another and to post-challenge grouping, two-dimensional hierarchical agglomerative clustering was performed on the 549 analytes after z-score normalization. In a manner consistent with the PAM/k-means clustering, hierarchical clustering clearly separated the pre- vs. post-SBP-Ag groups, and further identified two metabolomic populations in the post-SBP-Ag group (Figure 3A). One of these subgroups, including subject IDs 6, 11, and 9, clustered on the basis of metabolite expression but also represented those with a high abundance of eosinophils in the BALF (eosinophil number for each patient is annotated on the top). However, subject IDs 2 and 8 with a high abundance of eosinophils (EOS number in 200 µL BALF > 3 × 10^5^) were not clustered with subject IDs 9, 11, and 6. We next asked whether sufficient information was contained in the 72 identified metabolites; hierarchical clustering was performed using this subset. These metabolites also clustered the volunteers into the same two distinct SBP-Ag groups; subjects 6, 11, and 9 with the highest eosinophil numbers were distinct (Figure 3B). These findings indicated that information was contained in the identified groups that distinguished the SBP-Ag responses. 

### 2.4. Pathway Analysis

**Figure 4:**
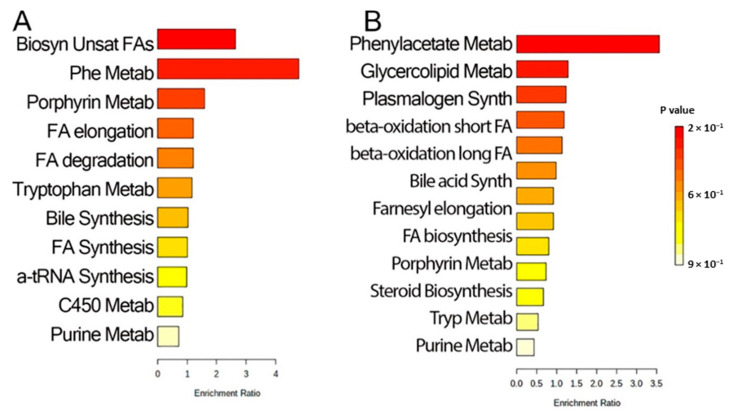
Pathway analysis. A total of 72 identified metabolites were analyzed for pathway enrichment in MetaboAnalyst 5. The bars represent the pathway ratio, representing the fraction of metabolites in the BALF data set relative to that of the entire pathway. Significance of enrichment is indicated by color, as indicated in the scale on the right. (**A**) KEGG library. (**B**) SMPDB library. Note that saturated fatty acid synthesis and metabolism are highly enriched in both analyses.

To obtain biological understanding, pathway analysis was conducted for the 72 discriminant metabolites. Ten pathways were identified in the Kyoto Encyclopedia of Genes and Genomes (KEGG) library, a library consisting of >15,000 compounds. The top-enriched pathways included (index metabolites indicated in parentheses): biosynthesis of unsaturated fatty acids (palmitic acid, stearic acid), phenylalanine metabolism (hippuric acid), porphyrin metabolism (bilirubin, chenodeoxycholate) and fatty acid elongation/degradation (palmitic acid) (Figure 4A). Similarly, pathways were enriched relative to the Small Molecule Pathway Database (SMPDB), a library containing > 40,000 compounds [24]. In a manner consistent with the KEGG library analysis, multiple representations of fatty acid biosynthesis, elongation, and metabolism were also found (Figure 4B). SMPDB pathways identified included phenylacetate metabolism (alpha-*N*-phenylacetyl-l-glutamine), glycerolipid metabolism (palmitic acid), plasmalogen synthesis (stearic acid), mitochondrial beta oxidation of saturated fatty acids (l-octanoylcarnitine), bile acid biosynthesis (chenodeoxycholic acid glycine conjugate) (Figure 4B). Collectively, these data identified a focal group of metabolites whose expressions were further examined.

### 2.5. SBP-Ag Induced Changes in Metabolite Expression Produces Two Classes of Response

From this analysis, 10 pathway-informing “index” metabolites that mapped to biologically relevant pathways whose pairwise changes in abundance was examined in response to SBP-Ag. These metabolites included l-tryptophan, whose abundance changed from 0.8 ± 0.3 to 2.9 ± 2.8 (these and subsequent values are given as median ± 25–75% IQR × 10^3^ intensity) after SBP. Octanoyl-l-carnitine increased from 4.9 ± 4.1 to 24.9 ± 36.5 after SBP. Hippurate went from 0.7 ± 0.6 to 2.4 ± 11.3. Phenylacetylglutamine changed from 0.99 ± 0.4 to 3.8 ± 7.4. Stearic acid increased from 63.7 ± 270 to 345.9 ± 475. Palmitic acid increased from 153 ± 386 to 488.6 ± 451.1. 

**Figure 5:**
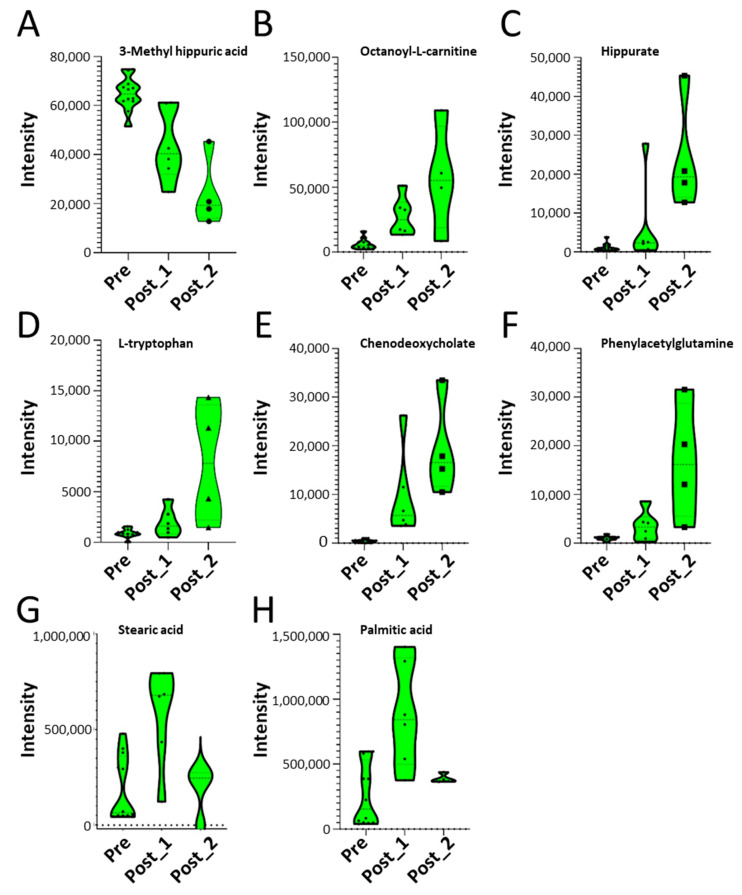
Abundance of signature metabolites in pathways. Shown are violin plots; the comparisons of intensities of major metabolites for the pre-challenge (Pre) or two distinct post-challenge samples. Each symbol is a BALF measurement: Pre, before SBP-Ag; Post_1, subject IDs 1, 2, 3, 4, 5, 7, 10, and 12 after SBP-Ag; Post_2, subject IDs 6, 8, 9, and 11 after SBP-Ag. Subject ID 8 was included in Post_2 subgroup because it was closer to the Post_2 subgroup in both PAM and k-means clustering. (**A**) 3-Methyl hippuric acid (C_10_H_11_NO_3_). (**B**) Octanoyl-l-carnitine (C_15_H_29_NO_4_). (**C**) Hippurate (C_9_H_9_NO_3_). (**D**) l-tryptophan (C_11_H_12_N_2_O_2_). (**E**) Chenodeoxycholate (C_26_H_43_NO_5_). (**F**) Phenylacetylglutamine (C_13_H_16_N_2_O_4_). (**G**) Stearic acid (C_18_H_36_O_2_). (**H**) Palmitic acid (C_16_H_32_O_2_).

To better visualize the differences between the two post metabolic phenotypes elicited by SBP-Ag challenge, the 10 index metabolites are plotted for each grouping (Figure 5A–G). Although these numbers are small, these data suggest distinct distributions of metabolite concentrations for the post-SBP subgroups as indicated by the violin contour estimation (Figure 5A–H).

### 2.6. Correlations of Metabolite Abundance with Cellular Inflammation

To better understand the complex relationships between the BALF metabolites and immune cell accumulation, a systematic correlation analysis was conducted. Here, the Pearson correlations of the abundance of the index metabolites and total cell counts in the same sample and the FCFeNO were calculated and presented as a correlogram. In this analysis, we grouped the two subgroups together to enhance power. 

**Figure 6:**
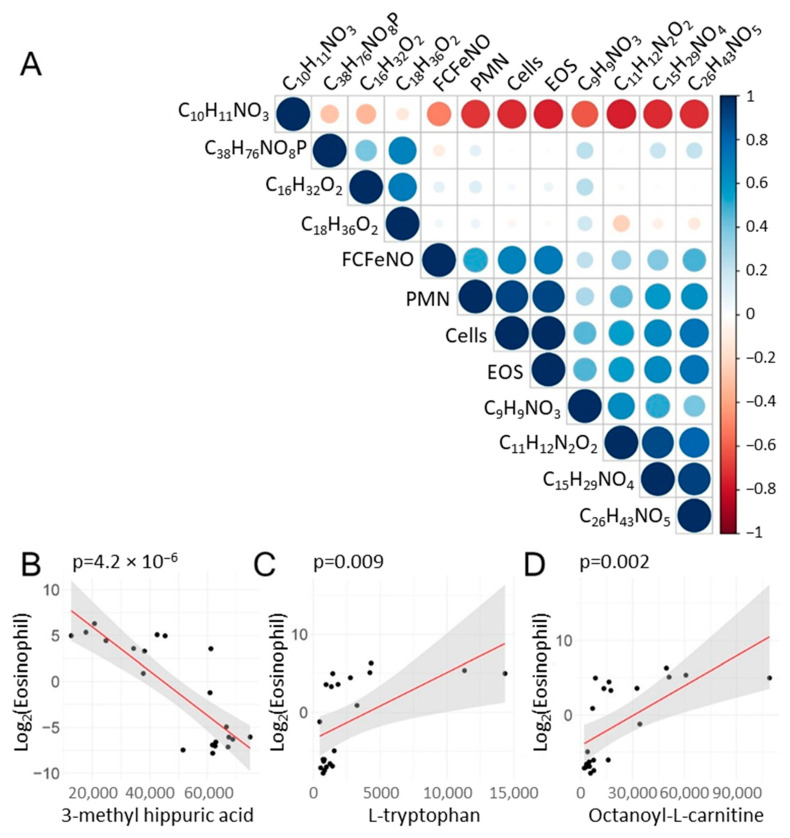
Correlation of metabolites and phenotypic features. Index metabolites were subjected to correlation analysis with BAL immune cell counts and FENO. (**A**) Correlation matrix correlogram from each sample (pre- and post-SBP-Ag) was used for the correlation. For each pairwise comparison, the Pearson correlation coefficient is indicated by the color scale (at right). Note the inverse correlation between 3-methyl hippuric acid (C_10_H_11_NO_3_) and leukocyte abundance (PMN, EOS, and total cells). In contrast, a strongly positive correlation was observed between C_11_H_12_N_2_O_2_ (l-Tryptophan) and eosinophil counts and a strong correlation between C_15_H_29_NO_4_ (octanoyl-L-carnitine) and eosinophil number. (**B**–**D**) Correlation plots of highly significant associations. (**B**) Linear regression modeling for Log_2_(Eos) vs. C_10_H_11_NO_3_ (3-methyl hippuric acid) abundance. Scatterplot and linear regression (in red) shown. Confidence interval of the regression is indicated by shading. Statistical significance (p) is indicated. (**C**) Log_2_(Eos) vs. C_11_H_12_N_2_O_2_ (l-tryptophan).

Consistent with the global analysis and that of the hierarchical clustering, the abundance of 3-methyl hippuric acid (C_10_H_11_NO_3_) was inversely correlated with eosinophil (Figure 6A). By contrast, tryptophan (C_11_H_12_N_2_O_2_) was positively correlated with eosinophil numbers as well as the octanoyl-l-carnitine (C_15_H_29_NO_4_) and palmitic acid (C_16_H_32_O_2_) (Figure 6A). 

The most significant correlations were subjected to linear regression after regularization of the eosinophil count by Log_2_ transformation. Highly statistically significant linear regression relationships were observed for the inverse relationship between 3-methyl hippuric acid (C_10_H_11_NO_3_) and Log_2_ transformed eosinophil number (Figure 6B), tryptophan (C_11_H_12_N_2_O_2_) and Log_2_ transformed eosinophil number (Figure 6C), and octanoyl-l-carnitine (C_15_H_29_NO_4_) with Log_2_ transformed eosinophil number (Figure 6D). 

### 2.7. Validation of Presence of Fatty Acid-Related Proteins in Blood Eosinophils

Because the changes in metabolites part of fatty acid pathways were correlated with the change in BAL eosinophil numbers, we analyzed the most abundant proteins present in blood eosinophils from subjects with allergies (see Supplement Methods and [25] for more details). In that previous publication [25], >5300 proteins were measured with relative abundances between 10.1 and 27.8. Here, we analyzed the 2000 most abundant of these proteins (abundance between 20.7 and 27.8) using DAVID Bioinformatic Resources 6.8 (Beta) (National Institute of Allergy and Infectious Diseases (NIAID), NIH) [26] to identify proteins part of the fatty acid pathway. We found four highly significant pathway related to fatty acid, including fatty acid metabolism (*p*(Benjamini corrected) = 1.2 × 10^−5^); fatty acid degradation (*p*(Benjamini corrected) = 1.4 × 10^−4^); and fatty acid beta-oxidation (*p*(Benjamini corrected) = 7.2 × 10^−4^) (Supplemental Table S4). A total of 39 proteins were part of these pathways. Therefore, these data confirm the tight association between the eosinophil and fatty acid metabolism that we identified by analysis of the BALF metabolites. 

## 3. Discussion

In this study, metabolomics profiling was applied to SBP-Ag to identify allergen-inducible phenotypes with the intent of elucidating potential endotypes of exacerbations. A striking finding is that unbiased associations of 549 metabolites identified at least two distinct metabolic profiles in response to the aeroallergen challenge that only emerged after the SBP-Ag challenge. From 72 identified metabolites, we identify the pathway signatures of fatty acid synthesis pathways, mitochondrial beta oxidation of saturated fatty acids, and other pathways relevant to asthma. From this pathway analysis, a discriminant group of 10 index metabolites were studied. These index metabolites show complex positive and negative relationships with each other, as well as measures of cellular inflammation. 

Previous studies using blood and urine metabolic profiling have largely focused on differentiating asthma as a disease vs. normal controls [27]. This is the first demonstration, to our knowledge, of an unbiased/data-dependent approach using metabolic profiling to identify allergen-inducible phenotypes in allergic asthma and offer potential approaches for elucidating disease-relevant endotypes. 

Our study focuses on the application of unbiased identification of inducible endotypes in acute asthma exacerbations. In support of this approach, we note that in the pre-challenge state, the resting abundance of metabolites are overlapping and do not discriminate between the response groups. By contrast, allergen challenge induces wide variations in metabolite abundance, indicated by the PCA, PAM, k-means clustering, and hierarchical clustering analysis. Importantly, these differences are not explainable by differences in the analyte collection since all patients were sampled under a standardized protocol. 

Substantial effort has been devoted toward identifying endotypes of asthma to inform precision treatments. Large scale cohorts, such as the SARP [12,13] and U-BIOPRED [14], have approached this problem using diverse sources of information, including age of onset, type of inflammation, exacerbators and presence of allergy, and/or remodeling to identify patients who may have similar endotypes [11]. However these clusters still contain substantial within-cluster heterogeneity, overlapping features [11], indicating that more work is needed to arrive at precision endotypes that are generalizable. 

In this study, we focused on the development of a rigorous platform to analyze BALF metabolites and provide a proof-of-principle that distinct metabolic outcomes can be generated by an allergen challenge. Here, we make the surprising finding that there are at least two metabolic outcomes of the allergen challenge; because this study cohort was restricted to mild–moderate asthmatics, we anticipate a greater number of metabolic phenotypes in the larger spectrum of allergic disease. We also note that a more robust clinical classification may be achieved by the integration of additional data types, including protein profiling, transcriptomics [28], blood based biomarker panels [29], and clinical features. 

Both KEGG and SMDB pathway analyses show that SBP-Ag induces changes in fatty acid metabolism, whose presence has been validated by blood eosinophil proteomic analyses. Previous mechanistic work has shown that fatty acid oxidation (FAO) enzymes significantly increased in a mouse model from OVA or HDM exposure [30]. In this model, HDM exposure enhanced the expression of the rate-limiting enzyme of the FAO pathway, carnitine palmitoyltransferase 1 (CPT1 or CPT1A), a pathway that regulates the entry of long-chain fatty acids into the mitochondria through converting acyl-CoAs to acylcarnitine derivatives. CPT1 expression is upregulated in the airway epithelium, neutrophils, and Siglec-F+ eosinophils, and its neutralization by a small molecule inhibitor reduced allergen induced reactivity [30]. Interestingly, inhibition of FAO reduced leukocytic inflammation, leading the investigators to propose that activated leukocytes require FAO to produce ATP. 

It is established that IL-5 and eosinophils are associated with the late phase response to an antigen and eosinophils induce ROS generation [31,32]. Defective mitochondrial beta oxidation of fatty acids has been linked to metabolic syndrome [33] and positively linked to asthma severity [34]. It will be of interest to examine dynamic changes in mitochondrial beta oxidation in future studies, controlling for levels of insulin resistance and body mass index. 

In this study, we further identified hippurate and 3-methyl hippuric acid as being index metabolites associated with a subgroup of high eosinophil responses. Previous work using nuclear magnetic resonance to measure exhaled breath condensates identified (and validated) hippurate as a discriminant marker of stable asthmatics versus normal controls [35]. However, this study did not examine the response to the aeroallergen challenge. Our work demonstrates this induction and relates hippuric acid metabolic pathways to eosinophils.

We note the presence of chenodeoxycholate (CDCA) in our index metabolites. Of interest, CDCA is a natural farnesoid X receptor (FXR) agonist and has been expressed in a mouse model of allergic airway inflammation, suggesting its role in normal and pathological lung physiology [36]. FXR functions as an antagonist to innate NF-κB signaling [37]. Interestingly, exogenous treatment with CDCA reduced OVA induced TNF secretion, cellular infiltration, goblet cell hyperplasia, and Th2 cytokine expression [36]. These findings suggest that some of the index metabolites may be involved in the resolution of inflammation. 

## 4. Materials and Methods

See Online Supplement for additional methods.

### 4.1. Segmental Bronchoprovocation with Allergen (SBP-Ag)

The University of Wisconsin–Madison Health Sciences Institutional Review Board (Madison, WI, USA) approved the study, and each participant provided written informed consent. As previously described [38], subjects had mild allergic asthma (aeroallergen skin prick test positive, FEV_1_ albuterol reversibility  ≥  12% or methacholine provocative concentration causing a 20% fall in FEV_1_  ≤  8 mg/,mL pre-albuterol FEV_1_  ≥  70%, and post-albuterol FEV_1_  ≥  80%) and none of the subjects were using inhaled or oral corticosteroids. Participants underwent the bronchial allergen challenge (BAC) to determine AgPD_20_, the allergen provocation dose resulting in a 20% reduction in FEV_1_ within 1 h of the challenge. Three allergens were used, including *Dermatophagoides farinae* (house dust mite), GS ragweed mix, or Fel d1 (cat) (all from Greer Labs, Lenoir, NC, USA). One month later, a baseline bronchoscopy with BAL was performed followed by SBP-Ag at a dose of 20% of each subject’s AgPD_20_. Forty-eight hours later, bronchoscopy with BAL was performed in the same challenged segment. The volume of saline for BAL was 160 mL and as an average ± SD, 115 ± 15 mL were recovered. A total of 14 subjects finished the SBP-Ag protocol and 2 were excluded for lower percentages of EOS in BAL after SBP-Ag (<15%). BAL cell differentials were determined by counting a total of 1000 cells on two cytospin preparations stained with the Wright–Giemsa-based Hema-3 (Thermo Fisher, Pittsburgh, PA, USA). Cell-free BALF were stored at −80 °C.

### 4.2. LC–MS/MS Analysis of BALF Samples

**Figure 7:**
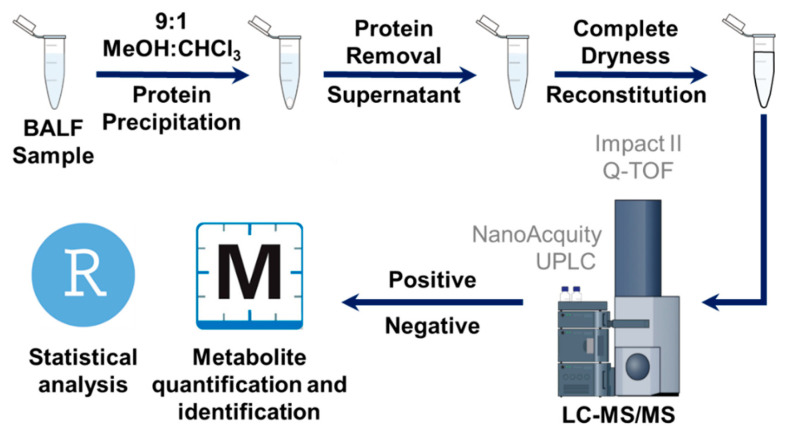
Sample workflow. Schematic view of sample preparation, data acquisition, and data analysis for BALF metabolomics.

LC–MS/MS experiments were performed using a Bruker Impact II quadrupole time-of-flight (QTOF) mass spectrometer (Bruker Daltonics, Bremen, Germany) coupled to a Waters nanoACQUITY UPLC system (Waters Corporation, Milford, MA, USA) using the workflow in Figure 7. The metabolites were loaded on a Waters nanoEase M/Z HSS T3 trap column (100 Å, 5 µm, 300 µm × 50 mm) for online desalting. A Waters nanoEase M/Z HSS T3 column (100 Å, 1.8 µm, 300 µm × 100 mm) was used for reversed-phase separation of the metabolites. The LC–MS/MS data were acquired in both positive and negative ion modes. For positive ion modes, mobile phases A and B were 0.1% formic acid in H_2_O and 0.1% formic acid in ACN, respectively. For negative ion modes, mobile phases A and B were 0.1% (*m*/*v*) ammonium bicarbonate in H_2_O and 0.1% (*m*/*v*) ammonium bicarbonate in ACN, respectively. The metabolites were trapped on the trap column for 5 min at 8 µL/min flow rate, and then were separated on the reversed-phase column at 4 µL/min flow rate using a 30 min stepwise gradient (99% A-0 min, 99% A-3 min, 1% A-10 min, 1% A-24 min, 99% A-24.2 min, 99% A-30 min). The column temperature was 40 °C. The metabolites eluted from the column were infused into the mass spectrometer using an electrospray ion (ESI) source. The end plate offset was 500 V, and the capillary voltage was 4500 V for the positive ion mode, and 3500 V for the negative ion mode. Nebulizer gas pressure was set to 0.5 Bar. Dry gas flow rate was 4.0 L/min, and dry gas temperature was 220 °C. MS/MS data were acquired in a data-dependent acquisition, dynamically choosing the top 10 precursor ions from the surveyor scan (MS) for collision-induced dissociation (CID). The same precursor ion was excluded after 2 spectra, and released after 0.3 min. 

### 4.3. Data Analysis

Bucket (mass) lists in the positive and negative modes were generated using the T-ReX 3D workflow in MetaboScape 4.0 (Bruker Daltonics, Bremen, Germany). The mzDelta was set to 0.50 mDa. The maximum charge state was set to 3, and the intensity threshold was set to 0. The minimum number of features for the result was set to 5. The bucket lists in the positive and negative modes were merged into one bucket list with 1.0 ppm *m*/*z* tolerance. Metabolites were identified by searching the LC–MS/MS data against the databases downloaded from MassBank of North America (MoNA), including NoNA-export-LipidBlast, NoNA-export-Experimental_Spectra, NoNA-export-HMDB, NoNA-export-MassBank, NoNA-export-LC–MS-MS_Spectra databases. Pathway analysis was performed using KEGG and SMPDB libraries. 

### 4.4. Statistical Analysis

Significant differences in analyte abundance were determined by an empiric Bayes approach using statistical analysis of microarray (SAM) [21]. In brief, SAM assigns a score to each analyte on the basis of change in metabolite expression relative to the standard deviation of repeated measurements. For metabolites with scores greater than an adjustable threshold, SAM uses permutations of the repeated measurements to estimate the false discover rate. In this study, statistical significance was adjusted, a delta of 1.0, and expressed as a “q-value”, adjusted for multiple hypothesis testing. The principal component analysis was in R (v.3.6). Hierarchical clustering involved using metabolic feature intensities in the pheatmap package (version 1.0.12) in R. Pathway enrichment of significant metabolites was performed in MetaboAnalyst 5.0 (https://www.metaboanalyst.ca/, accessed on 1 May 2021), and mapped to KEGG pathways. The matrix and graphical representation of Pearson’s correlations were calculated for metabolites and features by the corrplot library (version 0.92) in R. Log transformation of cell counts was used to satisfy parametric requirements. 

## 5. Limitations

Our study is limited by small numbers and limited numbers of metabolite identifications. Improved methods to enhance the number of metabolite identifications will extend the biological pathways. We also note that some index metabolites are components of multiple pathways; more improvements in pathway inference will enhance the application of metabolomics to provide actionable precision endotyping in the pathophysiology of asthma. As discussed earlier, we recognize that our study is limited to mild–moderate allergic asthma. Moreover, the correlative analyses performed at the peak of the inflammatory response used cell differential only, while inclusion of BAL mediators (cytokines, matrix proteins, etc.) would have been very informative to relate with the identified BAL metabolites.

## 6. Conclusions

Unbiased identification of inducible phenotypes in experimentally–induced asthma exacerbations was performed using high resolution MS of a segmental bronchial allergen challenge. We identified disease-relevant metabolic pathways, including saturated fatty acid synthesis, mitochondrial beta oxidation, and bile acid synthesis. 

## Figures and Tables

**Table 1:** Subjects’ demographics, *n* = 12 subjects.

Subject	Age	SBP-Ag	FEV_1_ (%)	Total BAL Cells (×10^4^ Cells/mL)	EOS (%)	PMN (%)	LYM (%)	MAC (%)	FeNO	Blood EOS/µL
1	23	Pre	75	14.8	1.1	1	3.6	94.3	56	229
		Post		145.4	74.5	4.8	6.9	13.8	82	510
2	27	Pre	102	10.4	0.4	0.2	2.5	96.9	19.9	387
		Post		233.7	73	1.9	2	23.1	30.1	493
3	31	Pre	135	7.7	0.5	1	4.8	93.7	41.4	290
		Post		17.9	51.7	5	7	36.3	59.8	290
4	36	Pre	87	8.9	0.4	0.1	4.8	94.7	77.1	185
		Post		68.3	72	1.9	6.8	20.2	66.3	405
5	21	Pre	83	11.1	0.2	0.4	7.2	92.2	49.9	343
		Post		76.5	77.2	1.7	3	18.1	66.8	519
6	27	Pre	95	7.1	0.4	1.2	8.6	89.8	40.4	255
		Post		221.7	71.3	3.2	6.6	18.9	57.1	510
7	27	Pre	71	6.4	1	1.8	14.5	82.7	47.9	167
		Post		90.7	66.3	5.4	8.6	19.7	57	132
8	19	Pre	103	12.5	0.6	0.9	5.3	93.2	24.5	387
		Post		193.7	80.6	1	4.8	13.6	69.2	942
9	22	Pre	108	38	0.2	0.7	32.2	66.9	23	202
		Post		542.6	72.7	3.6	14.2	9.5	52.5	396
10	27	Pre	83	11.3	ND	ND	ND	ND	28.7	70
		Post		12.2	17.7	9	12.4	60.9	55.6	325
11	20	Pre	100	8.6	0.6	0.7	8.8	89.9	49.4	316
		Post		263.5	77.8	1.4	12.2	9.6	99.3	941
12	33	Pre	99	9.1	ND	ND	ND	ND	ND	105
		Post		16.3	29.9	2.1	10.6	57.4	ND	140

Definition of abbreviations: Pre = pre-segmental bronchoprovocation with an allergen (SBP-Ag); Post = 48 h post-SBP-Ag; FEV_1_ (%) = forced exhaled volume in 1 s as % predicted; EOS (%) = percentage of eosinophils in BAL fluid; PMN (%) = percentage of neutrophils in BAL fluid; LYM (%) = percentage of lymphocytes in BAL fluid; MAC (%) = percentage of macrophages in BAL fluid; FeNO = fractional exhaled nitric oxide; ND = not determined.

## Data Availability

The data are available via ProteomeXchange with identifier PXD033172.

## Supplementary Materials

The following supporting information can be downloaded at: https://www.mdpi.com/article/10.3390/metabo12050381/s1, online supplement methods; Figure S1: Statistical Analysis of Microarray; Figure S2: Principal Components Analysis; Figure S3: Elbow plot; Supplemental Table S1: mass list; Supplemental Table S2: statistically significant metabolic features; Supplemental Table S3: statistically significant metabolite identifications; Supplemental Table S4: list of proteins part of fatty acid pathways.
