# Supplementary material for: Segmental Bronchial Allergen Challenge Elicits Distinct Metabolic Phenotypes in Allergic Asthma
Source: Metabolites. 2022 Apr 22;12(5):381. doi: 10.3390/metabo12050381 (PMC9145767; doi:10.3390/metabo12050381)
Supplement: Supplementary file 1 [file metabolites-12-00381-s001.zip › BALF Metabolites On_Line_Supplement_Methods.pdf]

Additional on-line Methods

Zhu et al, Segmental Bronchial Allergen Challenge Elicits Distinct Metabolic Phenotypes in Allergic Asthma

Materials

Reagents and chemicals were purchased from Sigma-Aldrich, Inc. (St. Louis, MO, USA) unless otherwise noted. HPLC-grade methanol, water and acetonitrile were purchased from Fisher Scientific (Fair Lawn, NJ, USA).

Sample Preparation and workflow

The workflow of sample preparation, data acquisition, and data analysis for BALF proteomics is shown in **Figure 7**. BALF samples were thawed on ice. Debris and cells were removed by centrifugation of BALF samples at 13,000 x *g* for 5 min at 4 ºC. 800 µL 9:1 methanol:chloroform was added into 200 µL BALF sample for protein precipitation. After vortexing for 10 s, the supernatant was removed by centrifugation at 15,000 x *g* for 10 min at 4 ºC, and was dried to complete dryness in a speed vacuum concentrator system. The dried sample was reconstituted in 100 µL H_2O for LC-MS/MS analysis.

Eosinophil preparation and proteomic analysis

Peripheral blood eosinophils were obtained from five allergic subjects with rhinitis with or without mild asthma and eosinophil blood counts between 229 and 308 per microliter (1) (Esnault J Proteome Research 2018). Subjects with prescriptions for low doses of inhaled corticosteroids did not use their corticosteroids the day of the blood draw. Eosinophils were purified by negative selection, as previously described. (Esnault S – J Proteome Research 2018). In brief, heparinized blood was diluted 1:1 in HBSS and was overlaid above Percoll (1.090 g/mL). After

centrifugation at 700*g* for 20 min at room temperature, the mononuclear cells were removed from the plasma/Percoll interface, and erythrocytes were eliminated from the cell pellet by hypotonic lysis. The remaining pellet was resuspended in 2% NCS in HBSS. Cells were then incubated with beads coupled to anti-CD16, anti-CD3, anti-CD14, and antiglycophorin-A (Miltenyi, San Diego, CA) and processed with an AutoMACS (Miltenyi). EOS preparations with purity >99% and viability ~98% were used. Eosinophils, 1×10^7, were cultured at 37 °C in 10 mL of medium (RPMI 1640 plus 10% fetal bovine serum) for 20 h. Cells were washed with PBS, snap-frozen in cold ethanol, and stored at −80 °C until all 5 samples had been obtained. Proteomic workflow and LC-MS/MS analyses were performed as previously described (Esnault S – J Proteomic Research). Protein abundance of the > 5300 proteins measured went from 10.1 up to 27.8. The top 2000 most abundant proteins were analyzed using DAVID Bioinformatic Resources 6.8(Beta) (National Institute of Allergy and Infectious Diseases (NIAID), NIH)(2). The mass spectrometry data have been deposited to the Chorus project (https://chorusproject.org), identifier 1464.

References:

1. **Esnault S, Hebert AS, Jarjour NN, Coon JJ, and Mosher DF**. Proteomic and Phosphoproteomic Changes Induced by Prolonged Activation of Human Eosinophils with IL-3. *Journal of Proteome Research* 17: 2102-2111, 2018.
2. **Huang DW, Sherman BT, and Lempicki RA**. Systematic and integrative analysis of large gene lists using DAVID bioinformatics resources. *NatProtocols* 4: 44-57, 2008.
