# Supplementary material for: Segmental Bronchial Allergen Challenge Elicits Distinct Metabolic Phenotypes in Allergic Asthma
Source: Metabolites. 2022 Apr 22;12(5):381. doi: 10.3390/metabo12050381 (PMC9145767; doi:10.3390/metabo12050381)
Supplement: Supplementary file 1 [file metabolites-12-00381-s001.zip › BALF Metabolites Supplemental Figures.pdf]

Supplemental Figures

Zhu et al., Segmental Bronchial Allergen Challenge Elicits Distinct Metabolic Phenotypes In Allergic Asthma

Figure S1. Statistical Analysis of Microarray. SAM plot of expected vs observed abundance of metabolites were plotted. Dashed lines are cut-off for for $\Delta=1.0$, corresponding to $Q<0.5$. Red dots are metabolites upregulated by SBP-Ag; green are downregulated.

Figure S2. Principal Components Analysis. Shown are two principal component dimensions (Dim) of PCA for subjects based on all metabolites measured. Note the close grouping of the pre antigen challenge samples, and the divergent post antigen challenge into two groups.

Figure S3. Elbow plot. Shown is an Elbow plot to determine the minimal cluster size for the K-means clustering. The most conservative number of groups (3) was used for K-means clustering.

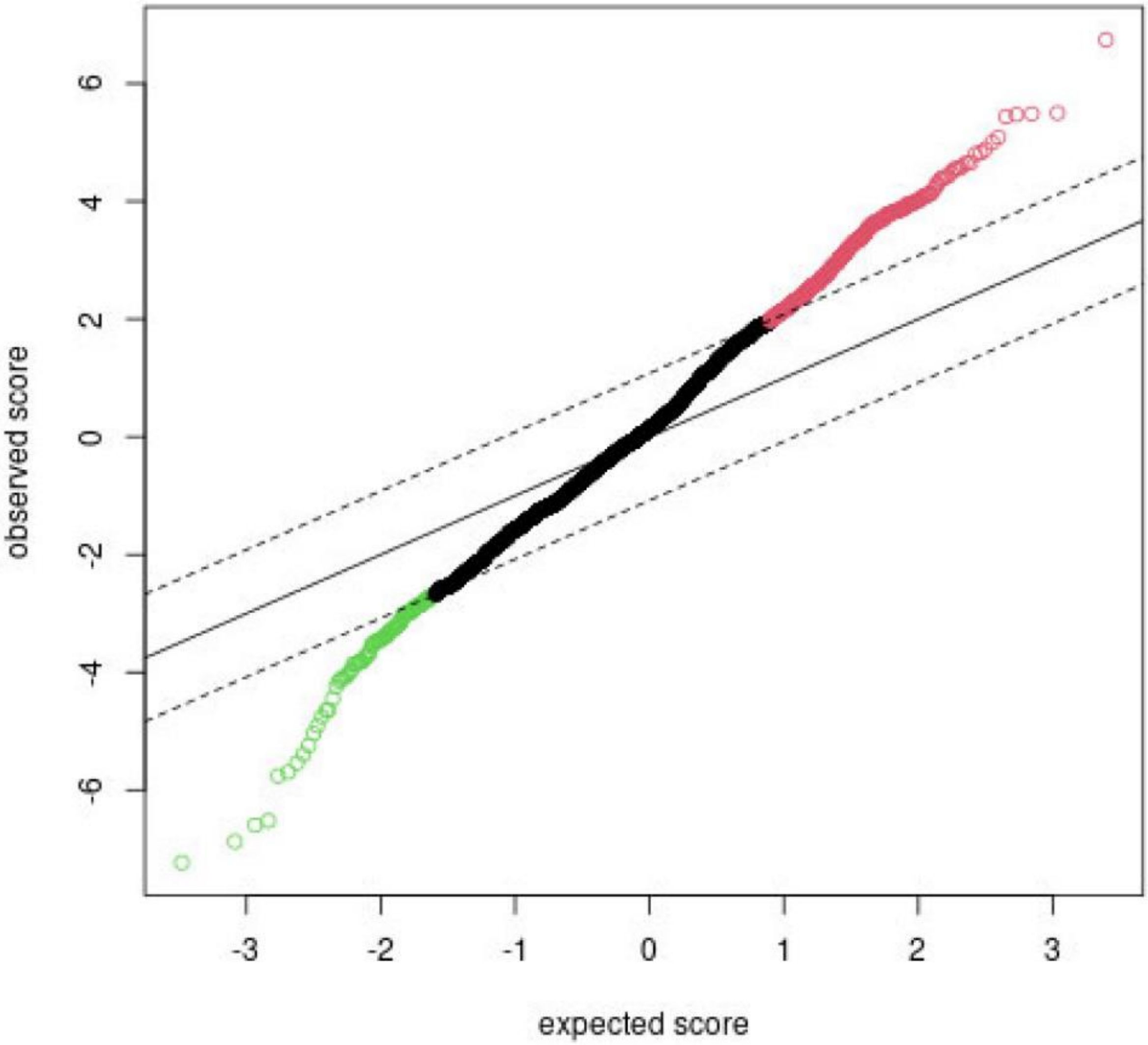

Figure S1. Statistical Analysis of Microarray. SAM plot of expected vs observed abundance of metabolites were plotted. Dashed lines are cut-off for for Δ=1.0, corresponding to Q<0.5. Red dots are metabolites upregulated by SBP-Ag; green are downregulated.

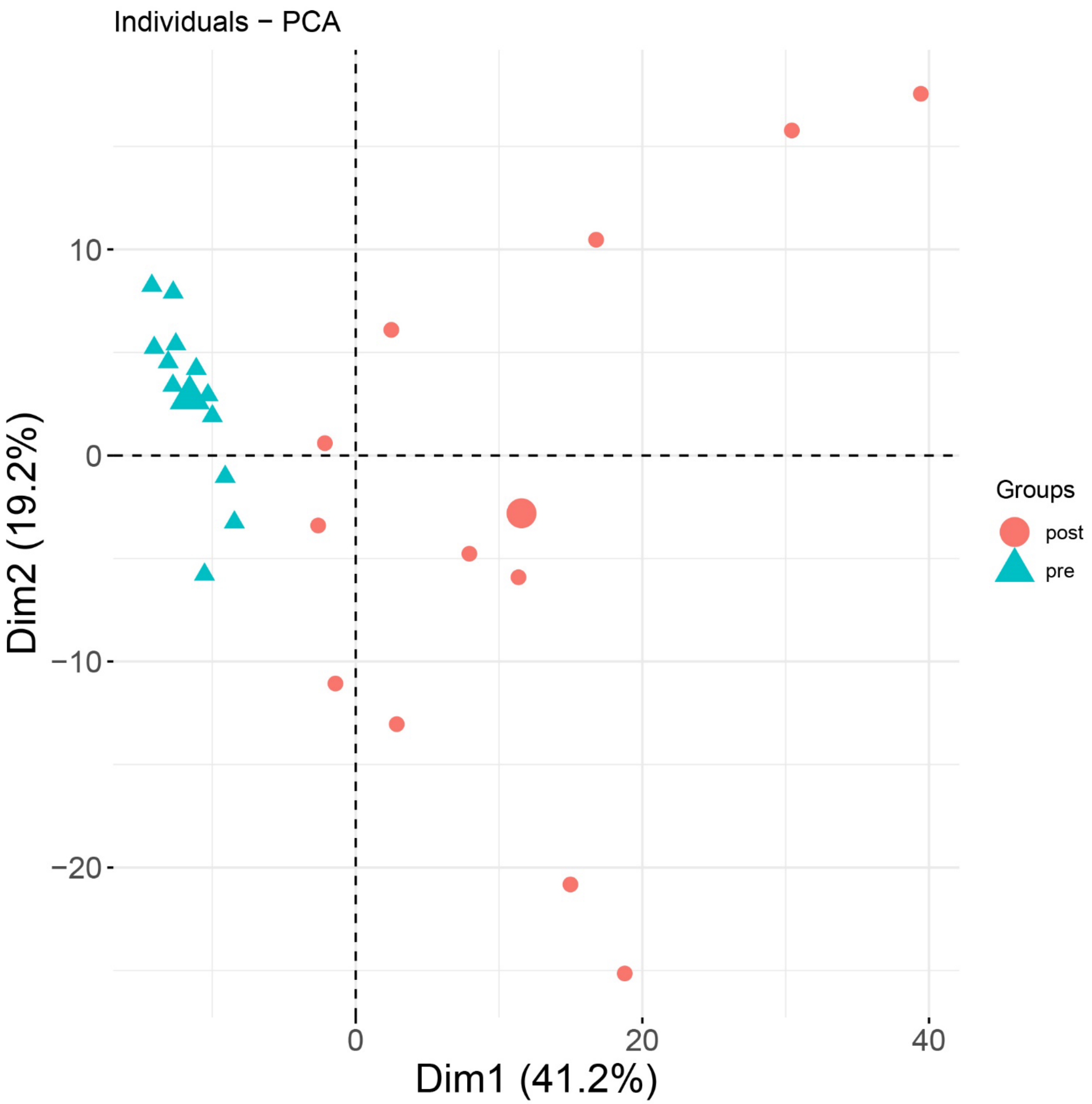

Figure S2. Principal Components Analysis. Shown are two principal component dimensions (Dim) of PCA for subjects based on all metabolites measured. Note the close grouping of the pre antigen challenge samples, and the divergent post antigen challenge into two groups.

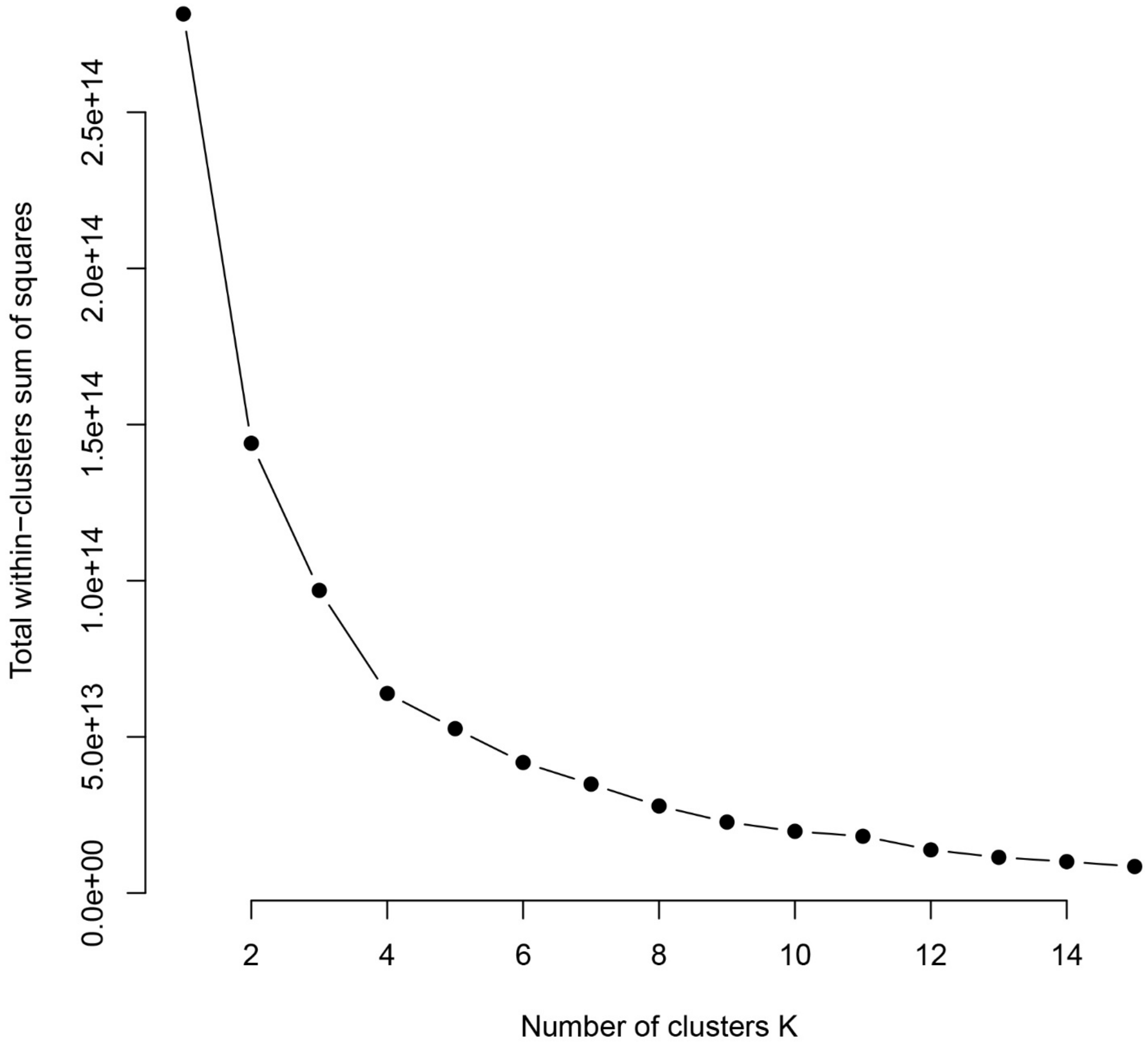

Figure S3. Elbow plot. Shown is an Elbow plot to determine the minimal cluster size for the K-means clustering. The most conservative number of groups (3) was used for K-means clustering.
