# Supplementary material for: Segmental Bronchial Allergen Challenge Elicits Distinct Metabolic Phenotypes in Allergic Asthma
Source: Metabolites. 2022 Apr 22;12(5):381. doi: 10.3390/metabo12050381 (PMC9145767; doi:10.3390/metabo12050381)
Supplement: Supplementary file 1 [file metabolites-12-00381-s001.zip › consent V13 clean 8-24-12 IRB approved.pdf]

Research Subject Information and Consent Form

Title of Study: Eosinophilic Airway Inflammation: Relationship to Remodeling and Modulation by Mepolizumab

Principal Investigator:
Nizar Jarjour, M.D.
University of Wisconsin
School of Medicine and Public Health
Madison Wisconsin

Invitation

You are invited to participate in a research study to test the effects of an investigational drug, called Mepolizumab, on the allergic cells in the airways of adults with allergic asthma. These cells are white blood cells and are called eosinophils. Your participation is completely voluntary. There is no anticipated medical benefit to you for participating in this study. You are invited because you are 18 to 50 years of age and have mild to moderate asthma that is triggered by an allergic reaction to house dust mite, ragweed or cat allergen. Approximately 40 subjects will participate in this study at our study center. We are the only study center conducting this study.

Summary

If you are eligible and decide to participate, you will receive the investigational drug called Mepolizumab. The drug is given as an infusion into a vein in your arm once a month for up to 3 doses during the study. It takes about 30 minutes to infuse the Mepolizumab. All of the eligible participants in the study will get Mepolizumab. This is called open-label.

The levels of eosinophils in your airways are measured during a procedure called a bronchoscopy. A bronchoscopy is an outpatient procedure where a small tube is inserted through your nose or mouth into your airway tubes in your lungs. We “wash” one of your airway tubes (segment) with a salt water solution. This washing is how we measure the levels of eosinophils. We will then stimulate one of your airway tubes (segment) during the bronchoscopy with a small amount of house dust mite, ragweed or cat allergen. This is called a segmental allergen challenge. Two days later you will return to the clinic for a second bronchoscopy. During this second bronchoscopy we will “wash” the segment that was exposed to the allergen. We will then measure the level of eosinophil in this washing.

About 4 weeks after your receive the Mepolizumab, you will have the 2 bronchoscopies, segmental allergen challenge and washings repeated. The level of eosinophil in the washing before Mepolizumab will be compared to the level of eosinophil in the washing after Mepolizumab.

You will come to the study site for at least 15 visits and up to 25 visits over 7-9 months. The visits will last for 1-10 hours. It may take more that one visit to complete the investigational drug treatment.

The study drug has not been approved by the U.S. Food and Drug Administration (FDA) and is available only for use in research studies.

Everyone in the study will have history and medication information collected, blood and urine tests, a genetic blood sample, breathing tests, allergy skin tests, sputum induction, methacholine challenges, physical exams, inhaled allergen challenges, bronchoscopies and segmental allergen challenges,. These procedures are discussed in detail in the following pages.

IRB Approval Date: 9/10/2012
Date IRB Approval Expires: 8/6/2013
FWA00005399 HS IRB
University of Wisconsin – Madison

The main risk of the study drug is potentially having an allergic reaction to it. We also don't know if it is dangerous for unborn babies so pregnant women may not be in the study. Side effects seen to date are rare and include headache, a skin rash, upper respiratory tract infection, muscle tenderness or pain, runny nose, sore throat, dizziness, itching, decreased ability to concentrate, tiredness, leg swelling, joint pain, nasal congestion and itching, nose bleed, productive cough, loss of hair, and bronchitis .

The main risk of the bronchoscopy procedure are sore throat, coughing, feeling tired, having a slight fever, and having more asthma symptoms for a day or two after the procedure. There is a very rare potential risk of serious complications.

You do not need to be in this research study to receive treatment for your asthma. If you decide to join the study, you can change your mind or stop at any time. If you complete the study, we will pay you approximately $1240. There will be no charge to you for any of the study related medicine, tests, or clinic visits.

More detailed information is on the following pages. If you have any questions, please call the principal investigator, Dr. Nizar Jarjour, at (608) 263-9344.

What is the Purpose of this Research Study?
The purpose of this study is to look at the effects of the study drug, Mepolizumab, on levels of a type of white blood cell, eosinophils, in your airways after you are exposed to allergen. Mepolizumab is being developed by GlaxoSmithKline and is a type of biologic drug called a monoclonal antibody that can block the actions of a protein molecule called interleukin 5 (IL-5). IL-5 is thought to increase the level of eosinophils in the blood or tissues. The levels of eosinophils in your airway after you are exposed to house dust mite, ragweed or cat allergen will be measured twice during the study. It will be measured before and after you receive Mepolizumab.

What will my participation involve?
If you decide to participate in this research study you will come to the study site for at least 15 visits and up to 25 visits over 7-9 months**.** (You may be required to come in for more than 1 visit to complete the investigational drug treatment.) There is a table attached at the end of this document showing what procedures are done during which visits. The visits will last from 1-10 hours. You will be contacted by phone two months after your last study drug infusion to collect information on your health and you will be asked to return to the research unit at 3 and 6 months after your last study drug infusion for follow up blood draws.
Screening –After reading and signing the informed consent, the following tests will be done to see if you are eligible to participate in this study. They will be scheduled over at least 3 visits.

- **Medical history:** We will ask you detailed questions about your past and present medical, surgical and medication history, asthma and allergy history, and smoking history.
- **Vital signs**: We will measure your temperature, blood pressure, how fast your heart is beating, and how fast you are breathing. We will put a clip on your finger that will measure your oxygen level in your blood, called pulse oximetry. We will measure your height and weight.
- **Urine test**: If you are a female of child bearing potential, you will also be given a pregnancy test several times throughout the study and following the study. You must agree to use a highly effective form of birth control with a failure rate of <1%, only have one male partner who is sterile, or you abstain from sexual intercourse while you are participating in this research study and for a period of three months after your last dose of study drug. If you have the potential to become pregnant, please discuss the form of birth control you are using or plan to use with your doctor before you begin receiving the study drug to ensure it meets these requirements. You will provide a small amount of urine for urine pregnancy testing if you are a female. A urine pregnancy test will be conducted twice during the screening period, on the

IRB Approval Date: 9/10/2012
Date IRB Approval Expires: 8/6/2013
FWA00005399 HS IRB
University of Wisconsin – Madison

days of a bronchoscopy procedure, and on the day of the study drug infusion as well as on your 3 month visit and 6 months after the last study drug infusion.

- **Allergy skin test**: Allergy skin testing is a common test to determine what allergies you may have. You will be tested for common allergens and 2 controls will be put on the underside of your forearm. Your skin will be lightly scratched where the drops of extract were placed. After 10-15 minutes we will look for redness and/or swelling (like a mosquito bite) where the tests were done. You may have localized itching at the site, and it may last for several hours.
- **Physical exam**: The study doctor will listen to your heart and lung, and look at your eyes, ears, nose and throat and do a general physical exam to ensure you are healthy.
- **Spirometry and reversibility**: This test measures how well your lungs are working. You will be asked to hold your albuterol for 6 hours if possible before each visit where spirometry will be measured. You will be asked to take a deep breath in and blow your breath out as hard and fast as you can into a machine for at least 6 seconds. This machine measures the amount of air you have in your lungs and how well you can blow the air out. We have you do this 2 or 3 times so we can get an accurate measure of your lung function. After spirometry you may be given 2 -4 puffs from an albuterol inhaler. We will wait 15 minutes and recheck your breathing to see how much it improves with medication. This is called reversibility.
- **Methacholine challenge**: This is a diagnostic test for asthma. If you have had one in the past you will not need to repeat the challenge. Methacholine is a chemical that can cause the airway tubes to constrict and can stimulate asthma symptoms. People who have asthma have airways that are sensitive to smaller concentrations of methacholine than people without asthma. This test measures how sensitive your airway tubes are to the methacholine. You will be asked to hold your albuterol for 6 hours if possible. We will measure your lung function. You will be asked to breathe in 5 puffs of methacholine, starting at a very low concentration. This will be given through a mouthpiece on a nebulizer. A nebulizer is a small machine that makes a mist you breathe in. Your lung function will be measured 3 minutes after each concentration of methacholine. If your lung function has not decreased by 20% the concentration of the next dose doubles. The challenge will be stopped when your lung function measurements have decreased 20% from your baseline or you receive the highest concentration we are using. This takes approximately one hour.
- **Whole Lung Allergen Challenge** You will be asked to hold your albuterol for 6 hours if possible. We will measure your lung function. You will be asked to breathe in 5 puffs of allergen, starting at a very low concentration. This will be given through a mouthpiece on a nebulizer. A nebulizer is a small machine that makes a mist you breathe in. Your lung function will be measured 10 minutes after each concentration of allergen. If your lung function has not decreased by 20% the concentration of the next dose increases. The challenge will be stopped when your lung function measurements have decreased 20% from your baseline or you receive the highest concentration we are using. This takes up to 2 hours. Your lung function will be measured at least every hour for 8 hours after the challenge is complete. You will be asked to measure your lung function after you leave the clinic with a hand held peak flow meter, and record it on a diary form. You will be asked to do this every hour until you go to bed, and again when you get up in the morning.
- **Blood tests**: We will do routine blood tests to check your health, see if you are pregnant, and make sure your blood clots normally. We will measure your eosinophil level and do blood studies in the research laboratory. We will check blood levels of study drug and antibodies to study drug. We will draw approximately 27 ounces of blood over the course of the whole study and you will have your blood drawn up to 27 different times. The amount of blood drawn over the course of this study equals around two American Red Cross donations.
- **Blood draw for genetics**: The purpose of the **genetics testing** is to take DNA from your blood cells to look at the variations in the a gene which controls the development of a pore (channel) on most white blood cells that helps to increase the cells ability to fight an infection and may help to control your bodies allergic response and genes related to asthma and inflammation.DNA is the material in human cells that carries genetic (inherited) information.

IRB Approval Date: 9/10/2012
Date IRB Approval Expires: 8/6/2013
FWA00005399 HS IRB
University of Wisconsin – Madison

Genetic information directs growth, development and how the body functions and there are many differences, or variations, in DNA from one person to another.

- **Exhaled nitric oxide (ENO)**: Nitric oxide is a gas that is released from inflammatory cells in the lung. We will have you gently blow air out into a machine for a 10 second period of time. ENO will be measured at several times before and after the inhaled and segmental allergen challenge.
- **Sputum Induction**: This is a procedure for obtaining some mucus or phlegm from your lower airways. Before the procedure you will be given 2 puffs of an albuterol inhaler to protect against bronchospasm (tightening of the bronchial tubes). You will inhale a mist of concentrated salt water through a mouthpiece for approximately 12 minutes. You will be asked to stop every 4 minutes, blow your nose, and rinse gargle and spit with water. Then you will be asked to cough up some of the mucus from your lower airways and spit it into a collection cup. We will check your lung function after every 4 minutes to make sure it hasn't decreased.
- **Bronchoscopy**: This is an outpatient procedure to obtain lower airway fluid, tissue and cells. You will come to the clinic after having had nothing to eat or drink for 12 hours. We will check your vital signs (blood pressure, pulse, breathing rate, and temperature). You will be asked to hold your albuterol for 6 hours if possible. We will measure your lung function. You will have breathing tests before and after 2 puffs of an albuterol inhaler. You will have a brief physical examination. You will get a shot in your arm of medicines that will help dry up your saliva, prevent your airway tubes from spasms, and relax you. Sometimes the medicine used to relax you will be given by a shot in the arm and sometimes you may get an IV (intravenous catheter). We will numb your nasal passages, throat and gag reflex with numbing medication called lidocaine. You will gargle this medication like mouthwash, inhale a mist of it, and have a gel that contains it placed in your nose. You will then have a small flexible tube introduced into your nose or mouth and passed through your vocal cords. We will use numbing medicine as we pass the tube into your lung. The tube will be wedged in one airway tube segment of your lung and we will put in 1 1/3 ounces of salt water and suction it back out again. We will repeat the washing 3 additional times. This is called a washing. The tube will then be wedged in a different airway tube segment of your lung and the washing repeated. This is how we obtain the lung fluid and cells to study. Bronchial brushing (removing cells from the lining of your lung with a soft brush) will be done during BAL#1 .We will then take 2 biopsies by pinching a piece of the lining of your airways with a wire called forceps. The bronchoscopy doctor may need to attempt the biopsy procedure up to 6 times in order to obtain 2 pieces that are usable. The bronchoscope tube will then be removed. We may videotape or take pictures through the scope when it is in your lungs. We will have you remain in the clinic until the medication we gave to relax you is worn off, your gag reflex has returned and you have eaten a meal. You will need to measure your peak flows every 2-4 hours after the procedure for the rest of the day. We will be providing hand held peak flow meters and will instruct you how to use it. If you receive relaxing medicine through an IV, you will need to stay in the research area for up to 8 hours and bring a relative or caregiver to help you to safety leave the research unit. The study staff will let you know if you need to bring a caregiver to help. We also call you 12 and 24 hours after your procedure for follow up. We require you remain in the Madison area for 24 hours after the bronchoscopy. The bronchoscopy will be done 2 days apart, 4 times during the study. One set (BAL #1 and #2) before the Mepolizumab Study Drug infusion, and one set (BAL #3 and #4) one month after the Mepolizumab Study Drug infusion.
- **Segmental Allergen Challenge:** This procedure will be done during a bronchoscopy. Two of your airway tubes will have about 1 teaspoon of allergen put in it while the scope is wedged in an airway tube segment. The allergen will stimulate this portion of your airway tube to produce eosinophils. The results of the whole lung inhaled allergen challenge will determine the dose given during this segmental allergen challenge. The scope will then be removed. Two days later you will return to the clinic. The bronchoscopy will be repeated to collect lung fluid and biopsy samples from the parts of the lung where the allergen solution was placed.

IRB Approval Date: 9/10/2012
Date IRB Approval Expires: 8/6/2013
FWA00005399 HS IRB
University of Wisconsin – Madison

- **Use of allergen extracts in Segmental Allergen Challenge:** The antigens that we use for the Segmental Allergen Challenge are commercially available and are FDA approved for use in allergy skin testing and allergy shots (immunotherapy). The use of these antigens in bronchoscopy is considered investigational. When making the antigen solutions to be used for challenge, we follow standard guidelines that have been published by asthma researchers.
- **Mepolizumab Study Drug infusion:** You will receive the study drug through a needle into a vein in your arm or hand. If you are a female and are able to have children, a urine pregnancy test will be done, and must be negative, before an infusion is given. The infusion will take approximately 30 minutes. The dose of the Mepolizumab will be 750 mg. You will receive at least one infusion, and no more than 3. You will receive infusions until the eosinophil count in your blood has decreased by at least 80% from your baseline. If your eosinophil count has decreased by at least 80% from your baseline, the second set of bronchoscopies will be done. The infusions will be approximately 4 weeks apart. You will have a weekly blood test after the infusions to measure your eosinophil count in your blood.
- **Anti- Mepolizumab Antibody and Mepolizumab Level Testing:** An antibody test is a blood test that looks to see if your blood is making antibodies against the study medication. If an antibody to the study medication does develop it may cause a reaction to the drug or be the reason that the drug is not effective. In all of the earlier mepolizumab studies, there were no antibodies seen in any of the patient's blood. The blood levels of mepolizumab will also be measured.

What are the risks of the study?
<u>Risks of Albuterol</u>: Common side effects are headache, increased pulse rate, shakiness of hands. Uncommon side effects are awareness of heart pounding or racing, mouth and throat irritation and muscle cramps. Rare side effects are low blood potassium, irregular heartbeat or heart rhythm, hyperactivity and an immediate increase in wheeze after dosing. Very rare side effects include an allergic reaction (hives, swelling of the face, mouth, tongue, and breathing problems). These are the most common side effects of these medications, but there are other less common events that are not listed. There may be side effects or risks that are unknown at this time.

<u>Risks of the Study Drug Mepolizumab</u>:
Mepolizumab is a type of biologic drug called a monoclonal antibody that can block the actions of a protein molecule called interleukin 5 (IL-5). IL-5 is thought to increase the level of eosinophils, a type of white blood cell, in the blood or tissues.

Based on data currently available from clinical studies, mepolizumab has been shown to reduce the numbers of eosinophils in the blood of patients with asthma and eczema in the absence of any major side effects. The safety profile of mepolizumab in these studies was similar to placebo. This means that the side effects seen in subjects taking mepolizumab were not significantly different than side effects seen in subjects taking an inactive substance like water (placebo). Other possibly related side effects that have been seen in studies and programs involving the use of mepolizumab include: headache, eczema (a skin rash), bronchitis (inflammation of the air passages), upper respiratory tract infection (common cold) and myalgia (muscle tenderness or pain), runny nose, sore throat, dizziness, pruritus (itching), decreased ability to concentrate, tiredness, leg swelling, peripheral edema (swelling of the ankles), arthralgia (joint pain), alopecia (loss of hair), epistaxis (nose bleed), productive cough, chest pain, vomiting, sinusitis (stuffy nose), nasopharyngitis (inflammation of the throat and nasal passage), asthma, wheezing, back pain, influenza, and oropharyngeal (mouth) pain and rhinitis (nasal congestion and itching).

As with any drug, there is a small chance that you will experience an allergic reaction to the medicine, such as an allergic skin rash, hives or possibly more serious problems such as breathing difficulties or shock. It is not possible to predict in advance if any of these problems will

IRB Approval Date: 9/10/2012
Date IRB Approval Expires: 8/6/2013
FWA00005399 HS IRB
University of Wisconsin – Madison

develop. You will be checked closely at regular intervals while you are receiving the study drug mepolizumab. If you have any severe allergic or infusion reactions, the infusion will be stopped and you will be given treatment to reduce the severity of these side effects. These reactions generally occur during the infusion or within hours after stopping the drip. The signs of these reactions may include rash, hives (red raised itchy bumps or rash), feeling sick (nausea), itching, erythema (redness of the skin) headache, flushing, sensitivity to light, mild chest tightness, and drop in blood pressure. Treatment is available if this occurs.

Studies show that mepolizumab is not thought to be involved in the development of cancers in patients. However, there have been a couple of reports of prostate cancer and a couple of reports of lymphoma, a type of cancer of white blood cells, in patients with a condition called hypereosinophilic syndrome (HES) who have received mepolizumab. However, some patients with HES have an increased chance of developing lymphoma because of the nature of the disease and mepolizumab is not thought to increase the chance of getting a lymphoma in patients with HES. As part of the general treatment for any patient who receives a monoclonal antibody, your doctor will monitor you for signs of lymphoma or any other cancer while you are receiving mepolizumab.

Risks of study procedures

- **Risks of holding albuterol:** You are asked to not use your albuterol inhaler for 6 hours before visits where spirometry will be measured. This includes all visits (methacholine challenge, and bronchoscopy) except Study Visit 2. You may experience increased asthma symptoms of shortness of breath, chest tightness or wheezing during this 6 hour time. If you are experiencing increased asthma symptoms during the 6 hour time of albuterol hold, please use your albuterol inhaler as needed and call the study coordinator before your appointment to let him/her know. It is possible your study appointment may need to be postponed. The study coordinator will be able to tell you if this is the case.
- **Allergy skin test:** You may itch at the puncture site, and it may last for several hours. In very rare situations skin testing can cause a severe allergic reaction. Should this occur, you will be treated with medication to reverse the reaction. You will need to remain in the office 20 minutes after the skin test is performed to make sure that no severe reaction is taking place.
- **Spirometry**: During spirometry you may feel short of breath during the 6-second exhalation measurement. If this occurs and does not go away on its own you may be given 2 puffs of an albuterol inhaler.
- **ENO:** There is no known risk associated with the exhaled nitric oxide procedure.
- **Sputum induction** is associated with chest tightness and a salty taste. You will be given 2 puffs from an albuterol inhaler before the sputum induction in order to prevent excessive chest tightness.
- **Methacholine challenge**: During this procedure you may experience asthma symptoms such as shortness of breath, wheezing, tightness or cough. You will be given 2 puffs of albuterol inhaler for relief of symptoms. The symptoms are usually gone within a few minutes after using the medication.
- **Blood draw and IV insertion**: You may experience some pain or bruising where the needle enters the skin. You may also have feel faint or sick to your stomach while having your blood drawn. There is a rare risk of infection where the needle enters the skin. The amount of blood drawn in this study may make you become anemic if you have low blood counts to begin with. Your blood counts will be measured at the screening visit and, if low, you will not be able to continue in this study. We will continue to measure your blood counts during routine testing throughout the study.
- **Whole Lung Allergen Challenge**. During the whole lung inhaled allergen challenge you are expected to have at least a 20% sustained decrease in your lung function. You may experience asthma symptoms such as chest tightness, shortness of breath, wheezing, and cough. These symptoms should be tolerable at rest. They are expected to last 30 to 60

IRB Approval Date: 9/10/2012
Date IRB Approval Expires: 8/6/2013
FWA00005399 HS IRB
University of Wisconsin – Madison

minutes and resolve on their own. If the response is significantly lower than expected, or you are reporting more symptoms than expected, we may give you inhaled albuterol to reverse the symptoms. You may have a second sustained decrease in your lung function during the 8 hour observation period after the challenge. This is called a late phase reaction. During the late phase reaction you may have a return of the asthma symptoms. In rare cases, the symptoms during the late phase reaction don't respond to the albuterol inhaler. If this happens, you will be given a dose of prednisone to take from the study doctor. Prednisone is a pill you swallow. The study doctor will prescribe how much and how often, usually only for a few days. The study doctor will also give you information on any side effects to expect at that time. The prednisone will be provided by the research clinic. If you have this rare response during the late phase you will not be eligible to continue in the study.

- **Bronchoscopy**: Under controlled situations (like in the clinic), the medical risks of a serious complication from bronchoscopy are low.
  In approximately 1 in 1,000 procedures a severe complication may occur that requires immediate medical attention. This includes severe bleeding, lung collapse, a severe allergic reaction to the anesthetic (numbing medicine), or a reaction to the medicine used to relax you.
  In approximately 1 in 100 procedures a moderate complication can occur. This can be a temporary worsening of your asthma symptoms, a high fever that develops after the bronchoscopy and lasts 24-48 hours.
  Midazolam or fentanyl, the medication given to relax you, may cause temporary side effects such as sleepiness, dizziness, unsteadiness and forgetfulness.
  In approximately 1 in 10 procedures a minor complication can occur such as sore throat from the tube for a day or two, short bouts of coughing which last 10-15 seconds as the numbing medication takes effect.
  Over the past 10 years there was one incidence of vocal cord irritation and swelling. This irritation can make it difficult to take a deep breath in, resulting in a feeling of shortness of breath. The symptoms may last for several days and are more likely to occur in subjects with acid reflux. Mild tiredness is common after the procedure. Brushing and biopsy adds little to the risk of bronchoscopy, except for a remote chance of increased bleeding. You may not feel the brushing, as your bronchial tube will be numb. When given in large doses the anesthetic used to numb your airways (lidocaine) may cause tingling, irregular heartbeats, allergic reaction or death. These reactions, however, are extremely rare. If you have a history of allergy to lidocaine, this procedure will not be done. The study staff will monitor the amount of lidocaine used during the bronchoscopy procedure very closely to insure that you do not receive too large of a dose.
- **Segmental Allergen Challenge:** Instilling antigen in the lungs can be associated with an acute asthma attack. This is a rare occurrence in our studies (less than 1 in 20 people) because we give all subjects an inhaled medicine, albuterol MDI, before the procedure, which is very effective in preventing these attacks. If you do get an asthma attack during the bronchoscopy, the procedure will be terminated and you will be given additional albuterol to relieve your symptoms. Furthermore, the individuals conducting the bronchoscopy have had extensive experience. There is a very remote chance (less than one in 100 people) that you could experience worsening asthma from the segmental allergen challenge after you leave the clinic. Using 2 puffs of your albuterol does not always relieve the worsening asthma symptoms. This is called a late phase response. You will be provided with a safety pack of prednisone with 24-hour phone number to call if you experience worsening asthma symptoms after you leave the clinic. The study doctor will at that time instruct you on how to take the prednisone tablets and what to expect. If you experience these late phase symptoms, or have other symptoms that persist 24 hours after the segmental allergen challenge, you will be asked to return to the clinic the next morning to be examined by the study doctor. You must return the prednisone safety pack at your next visit. The antigens that we use for the segmental allergen challenge are commercially available and are FDA approved for use in allergy skin testing and allergy shots (immunotherapy). The use of these antigens for segmental allergen challenge in bronchoscopy is considered investigational. We have done

IRB Approval Date: 9/10/2012
Date IRB Approval Expires: 8/6/2013
FWA00005399 HS IRB
University of Wisconsin – Madison

hundreds of segmental allergen challenges with these antigens over the last 20 years with no side effects other than what has been mentioned above.

- **Worsening asthma:** Your asthma may become worse during the study. The study staff will be reviewing how well your symptoms are controlled at every visit. If you are using more than 8 puffs per day of the albuterol inhaler, or have an increase in breathlessness with your usual activities, or have new symptoms during the study please call the study staff at (608) 263-0524 to speak with the research nurse. If your asthma becomes worse during the study you may require other medications. If this happens you will not be able to continue in the study.
- **Risk of disclosure:** Your research samples and data will not be labeled with your name or other personal identifiers. All data will be stored in a password protected, study specific database and will be labeled with a code number. Samples will be stored in one of Dr. Jarjour's research laboratories, labeled with a unique study code. There will be very limited access to the link between your name or other personal information and the study code. Because this link will exist, there is a remote risk that your name may become known in association with this research study.
- **Risk of disclosure for the genetic sample:** Procedures have been put into place that are designed to make it very difficult for the results from the genetic research to be linked to you. The DNA sample that you donate will be identified by a unique research study number. It will not be labeled with your name. The link that identifies you to this study code number will be kept completely separate from the study information and will be seen only by the study coordinator who does the study visit. The researchers doing the genetic testing will not be reporting the results as individual results but rather as a summary of all subjects tested. There is still a very remote chance that this information could accidentally become known to you, your doctor or others. If this happens it will not likely affect your health insurance or job because only asthma and inflammation related genes are being looked at. These are the best known risks of research in which genetic samples are used. The results of the genetic analysis will not be reported to you or your physician.

What are the benefits of the study?
You are not expected to benefit from participation in this study. The information gained from this study may provide information to help us better understand allergic disease and asthma and may also lead to new treatments.

Are there any costs?
The procedures done during the study are for research and are not standard medical care. The study medication and materials, study procedures, and study visits will be provided at no charge. All other aspects of your asthma care will be charged in the usual fashion.

Are there any alternatives?
You do not have to participate in this study to receive treatment for your asthma.

Will I be paid for my participation in the study?
Your participation in this study is voluntary and you will be paid up to $1240 if you complete the study procedures as below:

**Visit / procedures**	**Payment**	**Time involved**
Screening information, history, skin test, spirometry, reversibility	$25	1 ½ hours
Methacholine challenge with sputum induction	$35	1 ½ hours
Whole Lung Allergen Challenge	$100	10 hour
Sputum induction after Whole Lung Challenge	$25	1 hour
Bronchoscopy 1 and 3 with allergen challenge	$250	4-8 hour
Bronchoscopy 2 and 4	$200	4-8 hour
Blood draw visit before each study drug infusion	$15	15 minutes

IRB Approval Date: 9/10/2012
Date IRB Approval Expires: 8/6/2013
FWA00005399 HS IRB
University of Wisconsin – Madison

Visit / procedures	Payment	Time involved
Mepolizumab Study Drug Infusion visits	$50	1 hour
Weekly blood draw for eosinophil after study drug infusion	$15	15 minutes
Follow up visit at 3 and 6 months after study drug	$15	15 minutes

Will there be compensation for injury?
In the event that you are physically injured as a result of participating in this research, emergency care will be available. You will, however, be responsible for the charges for the emergency care. There is no commitment to provide any compensation for research-related injury. You should realize, however, that you have not released this institution from liability for negligence. Please contact the Dr. Jarjour at (608) 263-9344 if you are injured or for further information.

If I decide to start the study, can I change my mind?
Your decision to participate in this research is entirely voluntary. You may choose not to participate. If you do decide to participate, you may change your mind at any time without penalty or loss of benefits that you had prior to the study. You will be told of any new and significant findings, which may affect your willingness to continue. Your decision of whether or not to participate in this study will not affect the quality of your medical care at this institution.

The doctors doing this study may also decide that it is best that you leave the study.

If you leave the study early for any reason, regardless of whether it is the doctor's, or your decision, you will be encouraged to complete end of study procedures that include the safety assessments used in the study. These safety assessments may include blood and urine tests, physical exam, and breathing tests. You will be compensated $25 if you complete the visit and it will take approximately one hour.

<u>Residual Samples</u>
We will use the samples collected in this study for the scientific investigations described above. Occasionally the planned experiments do not consume the entire sample collected. Let us know whether the study investigator, Dr. Nizar Jarjour, may use the residual samples of your lung fluid and lung cells, tissue from the bronchoscopy or blood for future research related to respiratory disease and inflammation by selecting one of the following choices:

____ We may use your sample and the clinical study data associated with this sample for other research in our laboratory or share it with other investigators conducting approved research after removing all direct identifying information.
____ We may not use your sample and the clinical study data associated with this sample for any future research in our laboratory or share it with other investigators conducting approved research.

If you agree to have your residual samples used for future research, your samples will be stored until they are gone. This may take several months or several years. Your stored samples and data will be labeled with a code number, not your name. This code number is linked to your name in a protected computer file that is readable by only the study investigator and the direct study staff. Your samples will not be used for other genetic studies without your further approval. If you do not agree to the future use of your residual samples they will be destroyed.

Will my confidentiality be protected?
Your identity and medical records and data related to this study will be kept confidential, except as required by the law, and except for inspections by Agencies which regulate experimental drug

IRB Approval Date: 9/10/2012
Date IRB Approval Expires: 8/6/2013
FWA00005399 HS IRB
University of Wisconsin – Madison

studies, auditors, members of Institutional Review Boards or Ethics Committees. The UW Institute for Clinical and Translational Research's Data and Safety Monitoring Committee (DMC) may also review medical records and other information. Results of this research may be published for scientific purposes or presented to scientific groups; however, your identity will not be revealed. The data will be coded with a number. Only the Investigator and his study staff at this site have access to the code that links your name with your study number and data. Paper copies are kept in a locked file cabinet in locked rooms, electronic data is password protected.

During the study, you may need to go to doctor's office, clinics, or hospitals other than the study site for care related to your asthma. The study doctor or qualified personnel may request and review your medical records from these facilities.

What if I have questions?
If you have questions about this research, please contact the study investigator, Dr. Jarjour at 608-263-9344. If you have any questions about your rights as a research subject, contact UWHC Patient Relations Representative at 608-263-8009.

Authorization
I have read the information in this consent form, reviewed any questions, and I voluntarily agree to participate in this study. I have received a copy of this consent form.

______________________________ ____________________
Signature of Subject Date

____
Printed name of Subject

______________________________ ____________________
Signature of Person Date
Obtaining Informed Consent

IRB Approval Date: 9/10/2012
Date IRB Approval Expires: 8/6/2013
FWA00005399 HS IRB
University of Wisconsin – Madison

	Screening				Bronchs		Mepolizumab Dose 1					Bronchs		Follow-Up		
New visit #	1	2	3	4	5	6	7	8	9	10	11	22	23	24	25	26
							**Mepolizumab Dose 2 (optional)**									
							**12**	**13**	**14**	**15**	**16**					
							**Mepolizumab Dose 3 (optional)**									
							**17**	**18**	**19**	**20**	**21**					
**Days after last visit**		**>1**	**>1**	**2**	**>28**	**2**	**~21**	**<7**	**7**	**7**	**7**	**>21after drug**	**2**	**2 mon. after drug**	**3 mon. after drug**	**6 mon. after drug**
**Length of visit in hours**	**1 ½**	**1**	**10**	**1**	**4-8**	**4-8**	**½**	**1**	**¼**	**¼**	**¼**	**4-8**	**4-8**	**¼**	**¼**	**¼**
**Written informed consent**	**X**							**X**								
**Review eligibility criteria**	**X**	**X**	**X**		**X**	**X**	**X**	**X**			**X**	**X**	**X**			
**Medical history**	**X**															
**Medication history**	**X**															
**Vital signs**	**X**	**X**	**X**		**X**	**X**	**X**	**X**				**X**	**X**			
**Skin test**	**X**															
**Spirometry± reversibility**	**X**	**X**	**X**		**X**	**X**		**X**				**X**	**X**			
**Urine pregnancy test**	**X**		**X**		**X**	**X**	**X**	**X**				**X**	**X**		**X**	**X**
**Physical examination**			**X**		**X**	**X**		**X**				**X**	**X**			
**Methacholine challenge**		**X**														
**Whole lung inhaled allergen Challenge**			**X**													
**Sputum Induction**		**X**		**X**												
**Exhaled Nitric Oxide (ENO)**			**X**	**X**	**X**	**X**						**X**	**X**			
**Bronchoscopy**					**X**	**X**						**X**	**X**			
**Bronchial Brushing**					**X**											
**Segmental allergen challenge**					**X**							**X**				
**Study drug administration**								**X**								
**Blood safety (plt, pt/ptt, chemistry, cbc/diff) 12 ml**	**X**						**X**	**X**								
**Blood platelet only 4 ml**			**3X**	**X**	**2X**	**X**						**2X**	**X**			
**Blood eosinophil only 4 ml**									**X**	**X**	**X**	**X**			**X**	**X**
**Blood CBC diff**														**X**	**X**	**X**
**Blood studies (50 ml on V5&22, 280 ml on V6&23, 4ml on V3&4)**			**X**	**X**	**X**	**X**						**X**	**X**			
**Blood for genetics&pore assay (12 ml)**			**X**													
**Blood study drug levels and antibodies (14ml)**								**X**				**X**			**X**	**X**
**Assessment adverse events**		**X**	**X**	**X**	**X**	**X**	**X**	**X**	**X**	**X**	**X**	**X**	**X**	**X**	**X**	**X**
**Record concomitant meds**		**X**	**X**	**X**	**X**	**X**	**X**	**X**	**X**	**X**	**X**	**X**	**X**	**X**	**X**	**X**

IRB Approval Date: 9/10/2012
Date IRB Approval Expires: 8/6/2013
FWA00005399 HS IRB
University of Wisconsin – Madison
